# Toward Multiplexing Detection of Wound Healing Biomarkers on Porous Silicon Resonant Microcavities

**DOI:** 10.1002/advs.201500383

**Published:** 2016-02-04

**Authors:** Fransiska Sri Herwahyu Krismastuti, Alex Cavallaro, Beatriz Prieto‐Simon, Nicolas H. Voelcker

**Affiliations:** ^1^ ARC Centre of Excellence in Convergent Bio‐Nano Science and TechnologyFuture Industries InstituteUniversity of South AustraliaMawson Lakes, AdelaideSouth Australia5095Australia; ^2^ Future Industries InstituteUniversity of South AustraliaMawson LakesSouth Australia5095Australia

**Keywords:** confocal microscopy, diagnostic tool, fluorescence array, porous silicon resonant microcavity, sortase A

## Abstract

Bacterial wound infections can cause septicemia and lead to limb amputation or death. Therefore, early detection of bacteria is important in chronic wound management. Here, an optical biosensor based on porous silicon resonant microcavity (pSiRM) structure modified with fluorogenic peptide substrate is demonstrated to detect the presence of Sortase A (SrtA), a bacterial enzyme found in the cell membrane protein of *Staphylococcus aureus*. The combination of fluorescence enhancement effects of the pSiRM architecture with the incorporation of SrtA fluorogenic peptide substrate within the pSi matrix enables the sensing of SrtA with an outstanding limit of detection of 8 × 10^−14^ m. Modification of the pSiRM structure with microscale spots of two fluorogenic peptide substrates, one specific for SrtA and the other for matrix metalloproteinases, effectively demonstrates the feasibility to perform multiplexed biomarker analysis. The results in this study highlight the potential of the pSiRM sensing platform as a point‐of‐care diagnostic tool for biomarkers of bacterial wound infection.

## 1 Introduction

Managing chronic wounds constitutes a major health issue for the 21st century, and consumes significant amounts of healthcare funding.^1^ The presence of bacteria in chronic wounds is unavoidable, but certain bacteria and their biofilms delay wound healing and cause serious infections extending into the underlying bone or even systemic septicemia, with grave consequences for the patient.^2^ Two of the most common pathogens found in infected chronic wounds are *Pseudomonas aeruginosa* (*P. aeruginosa*) and *Staphylococcus aureus* (*S. aureus*).^3^

*S. aureus* is a Gram‐positive and catalase‐positive bacterium found in human skin and mucosal surfaces.^4^ *S. aureus* features a repertoire of virulence factors responsible for its survival during human host colonization and infection. These virulence factors are commonly displayed on the surface of the organism or secreted into the host milieu.^5^ *S. aureus* harnesses its virulence factors to escape from innate immune responses or promote tissue and cellular damage.^4^, ^5^, ^6^ It is thought that *S. aureus* exposure to host tissues beyond the skin or mucosal surface triggers upregulation of its virulence genes. As a result, *S. aureus* inoculation and biofilm formation into open wounds or damaged mucosal linings leads to infections of the respiratory tract and soft tissues.^7^

Sortase (Srt), a membrane‐anchored transpeptidase,^8^ is responsible for anchoring cell surface virulence factors involved in adhesion and virulence processes into the thick peptidoglycan layer of the host cell, and thus is essential for pathogenesis. The protein is important in bacterial survival during infections and has been identified in *S. aureus*[[qv: 8a]] as two enzyme isoforms, SrtA and SrtB, but only SrtA is expressed constitutively and has an important role to anchor surface proteins.[[qv: 8a,9]] Therefore, SrtA has been shown as a promising target for antibacterial therapy and for treatment of diseases caused by Gram‐positive bacterial infection including bacterial infection in chronic wounds.[[qv: 8a]] Nevertheless, to the best of our knowledge, SrtA has not yet been targeted as biomarker of bacterial infection. Biosensors able to detect SrtA at very low concentrations can offer great potential as diagnostic tools for bacterial wound infection.

*S. aureus* SrtA is a 206‐amino acid polypeptide with an N‐terminal signal peptide.[[qv: 8d]] SrtA cleaves surface protein sequences between the threonine (T) and the glycine (G) of the LPXTG motif (leucine, proline, X, threonine, glycine, where X is any amino acid)[[qv: 8b‐d]] and catalyzes the formation of an amide bond between the carboxyl group of threonine and the amino group of cell wall cross‐bridges.[[qv: 8b,c]] The ability to cleave the LPXTG motif is attributed to the nucleophilicity of the SrtA's active site, provided by a sulfur‐containing single cysteine residue at position 184.[[qv: 8c,10]]

**Figure 1:**
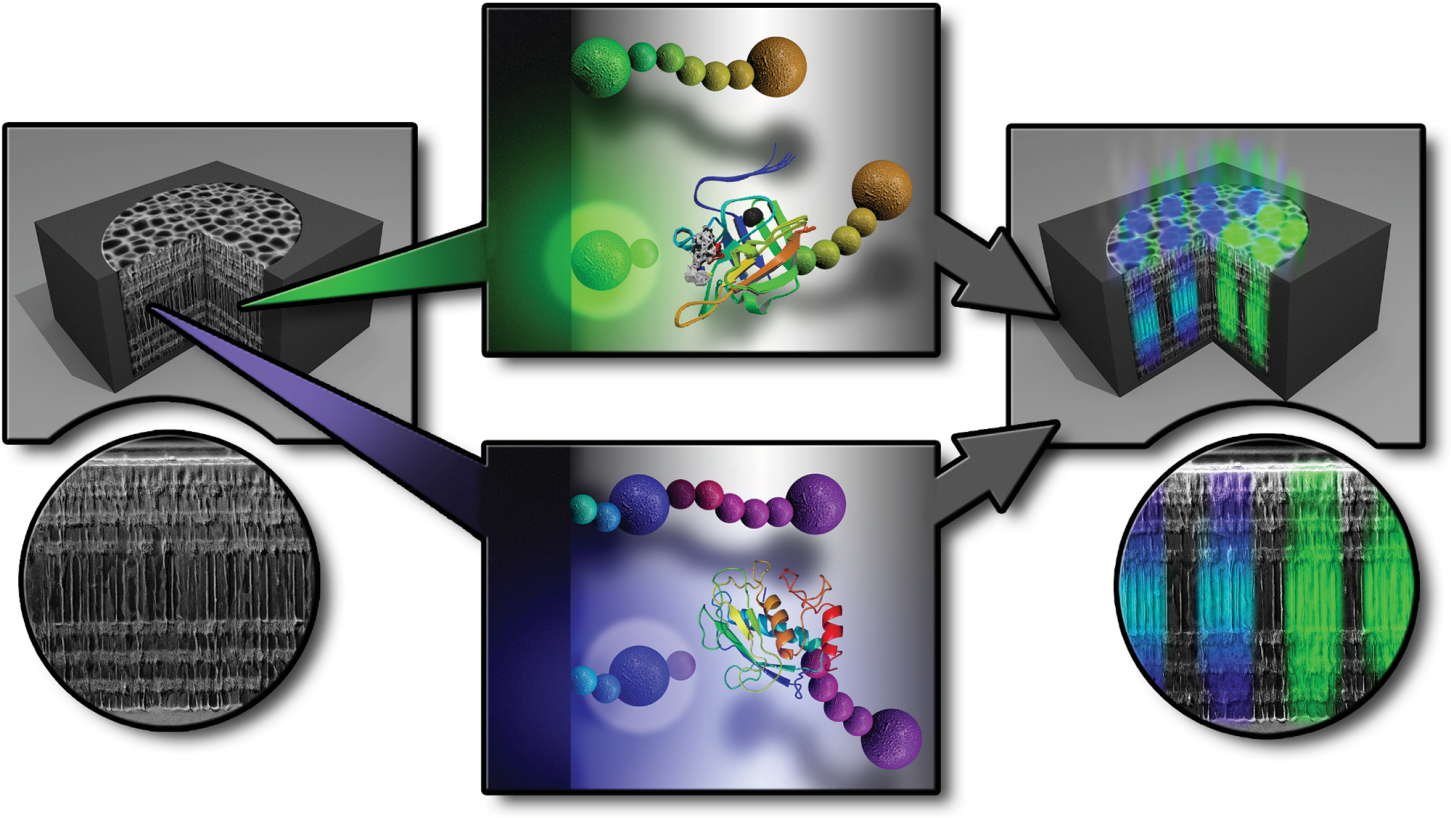
Schematic of multiplexed wound biomarker detection from a pSiRM (left) featuring an array of immobilized FRET substrates for SrtA (top center) and MMP (bottom center). Upon enzymatic cleavage (center panels), the confinement effect of light inside the cavity layer of pSiRM where the fluorophore is embedded induces a fluorescence enhancement effect (right).

In this study, we designed and developed a biosensor for SrtA to indirectly detect the presence of bacteria *S. aureus*. The biosensor was designed based on a porous silicon resonant microcavity (pSiRM) structure modified with a specific Förster (fluorescence) resonance energy transfer (FRET) peptide substrate containing the LPXTG motif. We used the SrtA substrate sequence, Dnp‐LPETG‐(K‐FITC)‐NH_2_, containing 2,4‐dinitrophenol (Dnp) as a quencher, fluorescein isothiocyanate (FITC) as a fluorescent dye, and an amino group to covalently anchor the substrate to the pores of the pSiRM. In the presence or SrtA, the peptide is cleaved and Dnp is removed, leaving FITC bound to the pSiRM. SrtA detection was performed in Hepes buffer solution and in complex samples, namely human wound fluid and bacterial culture medium. We also demonstrated the multi­plexing capability of the pSiRM sensing platform by detecting metalloproteinases (MMPs) and SrtA in parallel (**Figure** **1**).

## 2 Results and Discussion

### 2.1 Design, Fabrication and Characterization of pSiRM Sensing Platform

The sensing platform used to detect SrtA was based on a photonic pSiRM structure. The pSiRM structure has an active cavity layer in between two distributed Bragg reflectors (DBR) of alternating high porosity (HP) and low porosity (LP) layers. This architecture produces a characteristic optical spectrum with a narrow photonic resonance dip in the stop band of Bragg reflectors. The enhancement in photoluminescence from the pSiRM structure is based on a spontaneous emission of the emissive substance inside the active layer of the pSiRM, which is known as the Purcell effect.^11^ This effect is related to the local density of optical states in microcavities or plasmonic resonances.[[qv: 11b]] When pore size, porosity contrast, incident angle, and wavelength alignment of the photonic bandgap are tuned, sensitive biosensor platforms can be designed.

The pSiRM used in this study was based on our previously reported configuration of (HP/LP)_3_(HP)_4_(LP/HP)_3_.^12^ The HP layer was etched at a current density of 50 mA cm^−2^ for 2658 ms producing pore diameters ranging from 110 to 140 nm and 83.4% porosity, while the LP layer was etched at a current density of 25 mA cm^−2^ for 2237 ms resulting in pore diameters ranging from 40 to 60 nm and 67% porosity. These conditions were designed to accommodate SrtA molecules which have unit cell dimensions of 9 nm × 9 nm × 25 nm.^13^

The pore size and porosity contrast were chosen to optimize infiltration of the biomolecules and the sensitivity of the pSiRM sensing platform.^12^, ^14^ The pore size of both HP and LP layers has to be large enough to allow infiltration of SrtA, and the porosity contrast between HP and LP has to be considered in order to design a sensitive pSiRM biosensing platform.^12^ Those two parameters influence the effective refractive index, while the refractive index contrast between HP and LP layers affects the optical features of the pSiRM.^15^ In addition to that, the Q factor, which is defined as the ratio of the central resonance wavelength and the full width at half maximum (FWHM), is also crucial because it indicates the effectiveness of light confined in the active layer of the microcavity.^16^ Ouyang and Fauchet found that a high Q factor value indicates the more efficient light confinement inside of the microcavity, resulting in more effective fluorescence enhancement.^17^ However, based on our previous study, increasing the Q factor does not necessarily mean higher sensitivity of the biosensor.^12^

Our microcavity needed to produce a dip in the reflectance spectrum at a wavelength (λ) of 514 nm, aligned with the emission wavelength of the FITC dye. An aligned microcavity facilitates fluorescence emission enhancement of the fluorophore located within the pSiRM structure. To achieve that effect, we considered the wavelength shifts during the fabrication and surface modification steps. The reflectance spectrum of the pSiRM was first simulated using the transfer matrix method to obtain a dip at 514 nm with an angle of 36° in accordance with the angle during the fluorescence measurement set for maximum fluorescence emission.^12^ When this microcavity was fabricated as per the conditions from the simulation, the reflectance spectrum showed an 8 nm redshift. An additional 7 nm redshift was obtained after surface modification. Therefore, to compensate for those shifts and to produce the final wavelength of the microcavity dip at 514 nm after surface modification, the pSiRM was simulated for a dip located at 499 nm at a 36° angle.

**Figure 2:**
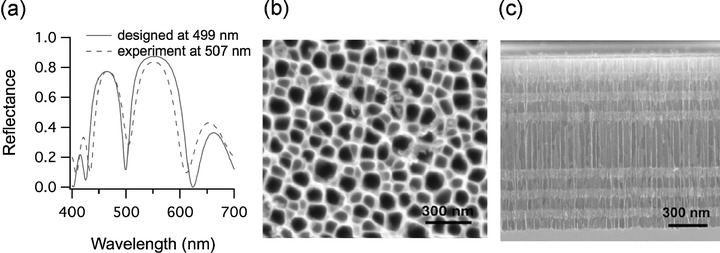
Characterization of pSiRM structure. a) Simulated reflectance spectrum of pSiRM (full line) and actual reflectance spectrum obtained using reflectance spectroscopy from freshly etched pSiRM (dashed line) measured at angle 36°. b) Top view and c) cross‐sectional SEM images of the freshly etched pSiRM.

In the simulation, the refractive indices (*n*) were 1.3 and 1.7 for the HP and LP layers, respectively. These *n* values combined with the *λ* of the designed microcavity dip were used to determine the thickness of each periodic layer considering the *λ*/4 rule for each DBR and *λ*/2 rule for the defect layer. This condition formed a 96 nm thick HP layer and a 73 nm thick LP layer. The total thickness of the pSiRM structure was 1.4 μm. Reflectance spectroscopy and scanning electron microscopy (SEM) were used to characterize the position of the microcavity dip and the morphology of the pSiRM, respectively (**Figure** **2**). The reflectance spectrum of freshly etched pSiRM (Figure 2a) shows that the position of the freshly etched microcavity dip was at 507 nm at an angle of 36° or redshifted about 8 nm from the simulation (499 nm). The top view (Figure 2b) SEM image shows that the pore diameter ranged from 110 to 140 nm (the first layer or HP layer) and the cross‐section (Figure 2c) SEM image shows that the thickness was around 1.4 μm, which is in agreement with the Scout simulation.

### 2.2 Surface Functionalization of the pSiRM

The modification of the pSiRM surface was essential for the stabilization and immobilization of peptide molecules on the pSiRM sensing platform.^18^ Here, hydrosilylation was used to modify the pSiRM.[[qv: 18b,19]] Thermal hydrosilylation of neat undecylenic acid on the pSiRM surface formed a dense alkyl monolayer with stable Si—C bonds, preventing the pSiRM surface from undergoing oxidative hydrolysis.[[qv: 19c,20]] The alkyl monolayer contained a distal carboxylic acid group, which can be activated in the form of succinimidyl esters to react with amino groups on the fluorogenic SrtA peptide substrate. The surface modification steps were monitored by means of FTIR spectroscopy in the attenuated total reflection (ATR) mode (Figure S1, Supporting Information).

The surface modification steps were also followed optically using reflectance spectroscopy to monitor the wavelength shift of the microcavity dip. After the hydrosilylation reaction, the position of the microcavity dip shifted 5.0 nm to longer wavelength (redshift). This shift was due to the additional long alkyl chain on the pSiRM surface. The activation of hydrosilylated surface using EDC/NHS also redshifted the dip position by 1.0 nm. These shifts were consistent with our previous results using the same surface modification reaction.^12^ The immobilization of SrtA substrate redshifted the microcavity dip by 1.2 nm. These gave a total redshift of 7.2 nm for the whole modification process.

### 2.3 Biosensor Principle

**Figure 3:**
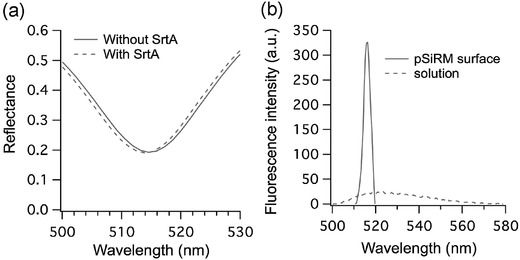
a) Reflectance spectra of pSiRM (zoom in the pSiRM dip) without SrtA (full line) and with SrtA (dashed line). b) Fluorescence emission from 1 × 10^−3^ m SrtA fluorogenic peptide in solution (dashed line) and immobilized on the pSiRM surface (full line) after incubation with 4.6 × 10^−8^ m SrtA in Hepes buffer.

Upon incubation with SrtA at 4.6 × 10^−8^ M, the position of the microcavity dip shifted 0.5 nm to the shorter wavelength (blueshift), indicating that a fraction of the peptide immobilized on the pSiRM surface was indeed cleaved (**Figure** **3**a). Thus, changes due to immobilization of SrtA substrate and its subsequent cleavage caused by Srt A gave a total shift of 6.7 nm which needs to be considered when designing the position of microcavity pSiRM. Note that for lower SrtA concentrations than 4.6 × 10^−8^ M, blueshifts in the reflectance spectra were not observable after incubation of the pSiRM.

We thus investigated if FITC fluorescence from the pSiRM surface upon cleavage of the Dnp quencher and the fluorescence enhancement effects in the aligned pSiRM would translate into improved sensitivity of detection.^21^

The SrtA peptide‐modified pSiRM surface was incubated with 4.6 × 10^−8^ m SrtA in Hepes buffer solution. To confirm the fluorescence enhancement effect of the pSiRM, a similar experiment was also performed in the solution phase, where 1 × 10^−3^ m SrtA peptide solution was incubated with the same concentration of SrtA (Figure 3b). As further control experiments, the SrtA substrate‐modified pSiRM and the SrtA substrate solution were incubated in the absence of SrtA for the same incubation time.

The strong FITC emission after SrtA cleavage of the SrtA substrate immobilized on the pSiRM surface was 13‐fold higher than that detected upon cleavage of the SrtA substrate in solution (Figure 3b). In addition to that, the emission peak of the FITC dye in solution (FWHM ≈ 36 nm) was nine times broader than the FITC emission peak from the pSiRM surface (FWHM ≈ 4 nm). The fluorescence emission from different pSi structures (single layer with LP, single layer with HP and multilayer) and untuned pSi were compared (Figure S2, Supporting Information). Taken together, our results confirm the fluorescence enhancement effect of the pSiRM sensing platform and the confinement effect of light that escapes from the microcavity surface.^21^, ^22^

### 2.4 Biosensor Performance

The performance characteristics of the developed biosensor were investigated. First, studies of the effect of the incubation time on fluorescence emission signal were performed. The incubation of 4.6 × 10^−8^ m SrtA on the SrtA peptide‐modified pSiRM with the tuned cavity wavelength (Figure S3, Supporting Information) showed that after 5 min incubation time, a conspicuous fluorescence emission signal appeared, indicating that the SrtA substrate was already being cleaved and reached a maximum with incubation time up to 30 min, which is in agreement with previously reported results.^23^ Therefore, further experiments were performed with an incubation time of 30 min.

The measured fluorescence intensity increased linearly with increasing concentration of SrtA enzyme from 4.6 × 10^−12^ m to 4.6 × 10^−8^ m (four orders of magnitude) with a linear regression equation of y = 57 *x* + 744 (*R* ^2^ = 0.9997) (Figure S4, Supporting Information). The lowest SrtA concentration tested was 4.6 × 10^−18^ m. The limits of detection (LOD) and quantitation (LOQ), calculated as y_b_ + 3Std_b_ and y_b_ + 10 Std_b_, respectively, where y_b_ is the fluorescence measured for the blank (control solution in the absence of SrtA) and Std_b_ is the standard deviation of blank, were 8.0 × 10^−14^ m and 8.3 × 10^−14^ m, respectively. This result confirms that the fluorescence‐based sensing approach by far exceeds the performance of the reflectance‐based sensor which gave a LOD in the order of 10^−8^ m. Moreover, the fluorescence‐based pSiRM sensor outperforms the quantitative immuno‐PCR assay for SrtA in terms of LOD (10^−14^ m vs 10^−12^ m) and analysis time (30 min vs 4 h including the PCR step),^24^ demonstrating its potential as a point‐of‐care diagnostic tool.

### 2.5 SrtA Detection in Human Wound Fluid and Bacterial Culture

**Figure 4:**
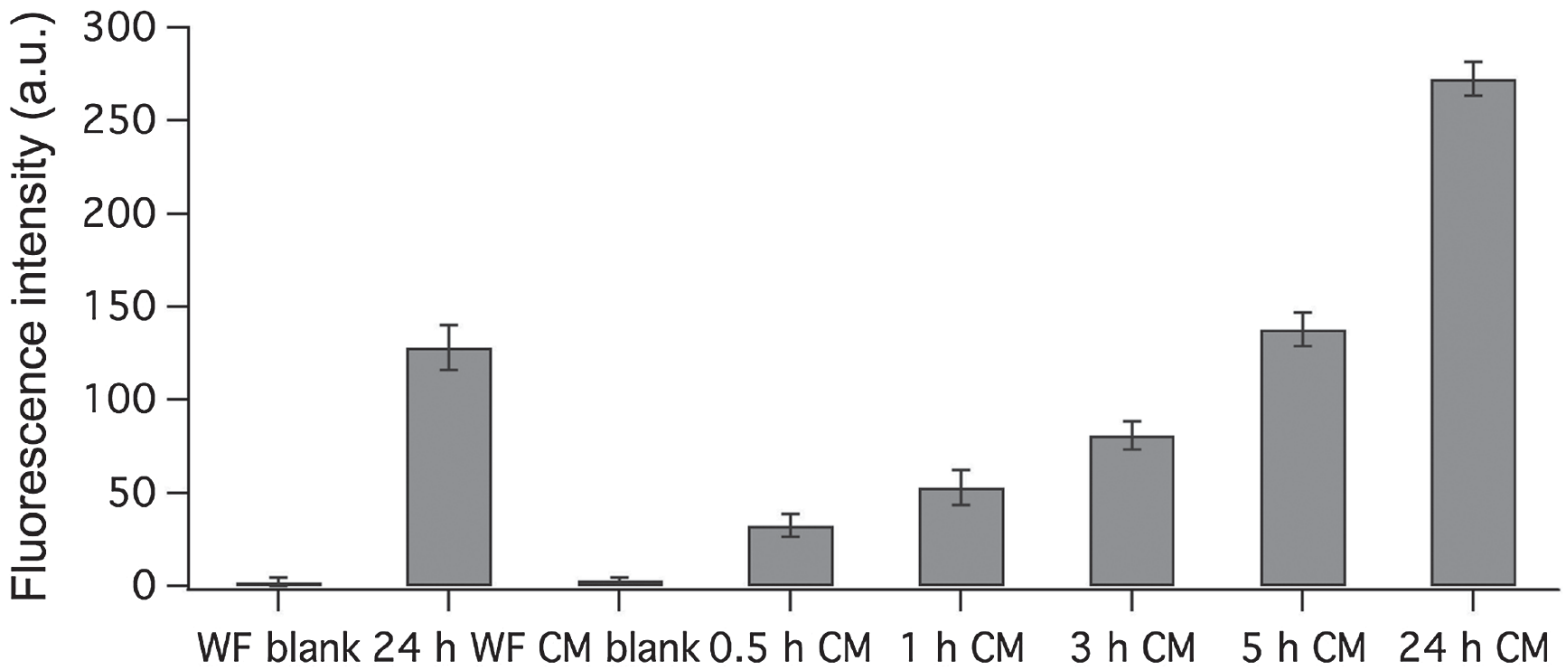
Fluorescence intensity from the SrtA fluorogenic peptide substrate‐modified pSiRM after incubation with wound fluid that had been inoculated with *S. aureus* for 0 and 24 h and then filtered, or incubation with bacteria culture medium (CM) inoculated with *S. aureus* for 0, 0.5, 1, 3, 5, and 24 h. Error bars were calculated from three independent experiments.

We also demonstrated the capability of the pSiRM sensing platform to detect the presence of SrtA in complex biological samples, such as human chronic wound fluid and bacterial culture medium (**Figure** **4**; Figures S5 and S6, Supporting Information). Here, we used human wound fluid, human wound fluid inoculated with *S. aureus* for 24 h, bacterial culture medium and bacterial culture medium inoculated with *S. aureus* at different times (0.5, 1, 3, 5, and 24 h) to study the effect of the inoculation time on the intensity of the fluorescence signal (Figure 4). A clear fluorescence signal was already observed from a pSiRM sensing platform incubated with bacterial culture medium after 0.5 h inoculation with *S. aureus* corresponding to a bacteria concentration of 1.3 × 10^8^ CFU mL^−1^. Concentration of *S. aureus* increased with inoculation time: for 1, 3, 5, and 24 h inoculation times, the concentration of *S. aureus* was 2.1 × 10^8^, 5.1 × 10^8^, 1.1 × 10^9^, and 2.6 × 10^9^ CFU mL^−1^, respectively. Incubating the solutions with increasing bacteria concentrations on the pSiRM gave rise to increasing fluorescence intensity. This was expected since *S. aureus* growth leads to increased SrtA concentration in wound bed as observed by Yarets et al.^25^

FITC fluorescence emission was also detected in human wound fluid inoculated for 24 h with *S. aureus* and then filtered. Comparing wound fluid and bacterial culture medium for the same inoculation time, the wound fluid sample showed lower fluorescence intensity, indicating that the bacterial growth in the culture medium is faster than in the wound fluid. This is expected since the culture medium has optimum conditions for bacterial growth. However, our sensing platform was still able to detect SrtA directly in the wound fluid sample.

**Figure 5:**
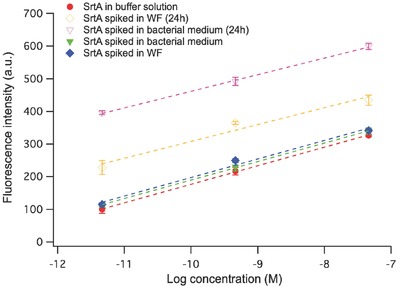
Fluorescence emission intensity from the SrtA fluorogenic peptide‐modified pSiRM after incubation with the same concentrations of SrtA in Hepes buffer solution (red dot), human wound fluid (blue diamond), bacterial culture medium (green triangle), wound fluid inoculated with *S. aureus* for 24 h (yellow circle), and bacterial culture medium inoculated with *S. aureus* for 24 h (pink triangle). Error bars were calculated from three independent experiments.

We also studied matrix effects that may be caused by other biomolecules in wound fluid or bacterial culture medium. Fluorescence emission of human wound fluid and bacterial media, with and without *S. aureus* (24 h inoculation time) was determined after spiking with 4.6 × 10^−8^ m, 4.6 × 10^−10^ m and 4.6 × 10^−12^ m SrtA (**Figure** **5**).

The fluorescence signal generated on the pSiRM samples exposed to either wound fluid, bacterial culture media, wound fluid inoculated with *S. aureus* for 24 h, or bacterial culture medium inoculated for 24 h containing SrtA showed linear relationships with the concentration of SrtA. The percentage of slope deviation between calibration curves for each one of the biological samples and for spiked buffer samples was in all cases less than 1%, indicating the absence of matrix effect. The offset of the lines is related to the initial SrtA concentration in each complex matrix (wound fluid and bacterial culture medium with and without 24 h *S. aureus* inoculation).

The biosensor described here has the potential to become a diagnostic tool of wound status affording rapid medical assessment that can be used to guide treatment actions, such as adding topical antibiotics in response to the detection of bacterial infection. In particular, this tool could be deployed as a point‐of‐care diagnostic device of wound exudates able to provide fast, sensitive, and selective response of *S. aureus* infection. Recently, we reported a MMP biosensor for the detection of MMP‐1 also based on the fluorescence enhancement effect achieved using pSiRM.^12^ MMP‐1 is produced by cells approximately 1 week after wounding or at the postacute stage of healing.^26^ While the MMP biosensor that we recently reported provides accurate information by monitoring the changes in the levels of MMP‐1 after wounding and during the healing process, our SrtA biosensor confirms bacterial infection by means of discrete detection of this bacterial enzyme. In addition, our study is the first demonstration of multiplexed detection, which is expected to translate into a biosensor that it useful from a clinical perspective since it can distinguish different types of enzymes present in the wound environment.

### 2.6 Fluorescence Detection Using Confocal Microscopy

To demonstrate that fluorescence microscopy can be used to qualitatively observe the presence of SrtA on the pSiRM sensing platform, one half of the pSiRM sample was functionalized with SrtA peptide substrate (sample surface) and the other was left unmodified. This was achieved by masking with PDMS during surface functionalization.

**Figure 6:**
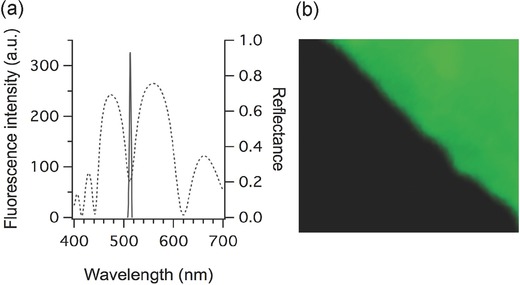
Fluorescence emission from the SrtA fluorogenic peptide‐modified pSiRM detected using a) fluorometer and b) confocal microscopy. The dashed line in panel (a) is the reflectance spectrum of the pSiRM while the full line is the FITC emission. The confocal microscopy image was taken from the SrtA fluorogenic peptide substrate‐modified pSiRM on one half of the pSiRM sample (green area) and the control surface (black area) which was masked during the modification steps. The surface was incubated with SrtA in Hepes buffer for 30 min before imaging.

The confocal microscopy image in **Figure** **6**b shows the boundary between the regions with and without peptide substrate upon exposure to SrtA as green‐fluorescing (FITC intensity emission shown in Figure 6a) and dark, respectively.

**Figure 7:**
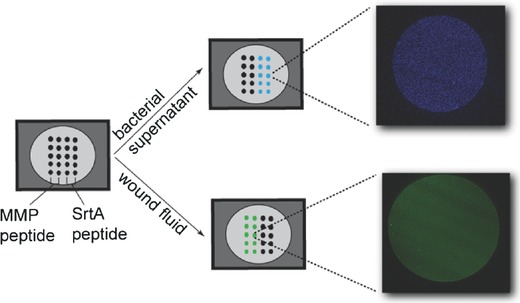
Confocal microscopy images of the fluorescence emission from the fluorogenic SrtA peptide substrate immobilized on the pSiRM after incubation with bacterial culture medium (top) and fluorescence emission from the MMP fluorogenic peptide immobilized on the pSiRM after incubation with human wound fluid (bottom).

On the basis of this result, an array of peptide spots was produced on a pSiRM sample via robotic printing of two different fluorogenic peptide substrates to explore the multiplexing possibility of the biosensor. One peptide was specific for MMPs and the other for SrtA. The peptide substrates were printed on the activated pSiRM surface following the pattern shown in **Figure** **7** featuring spots of 350 μm diameter. The array on the pSiRM surface was incubated with human wound fluid and bacterial culture medium samples, respectively. Note that the presence of MMP‐1 in the wound fluid sample was confirmed using Western blotting.^27^

The pSiRM surface incubated with wound fluid showed only blue fluorescence emitted from the rows of MMP peptide substrate spots corresponding to the emission of EDANS dye upon cleavage of the MMP‐specific peptide substrate with the sequence Dabcyl‐Gaba‐Pro‐Gln‐Gly‐Leu‐Glu(EDANS)‐Ala‐Lys‐NH_2_. These confocal images corroborate the presence of MMPs and the absence of SrtA in the wound fluid sample. The pSiRM surface incubated with bacterial culture medium after 24 h inoculation showed green FITC fluorescence emitted from the rows containing SrtA peptide substrate spots. These experiments corroborate that the SrtA present in bacterial culture medium sample did not cleave the MMP peptide substrate and vice versa, and effectively demonstrate the selectivity of the peptide substrate immobilized on the pSiRM.

## 3 Conclusion

In conclusion, we have demonstrated the detection of SrtA bacterial enzyme by utilizing a pSiRM modified with a fluorogenic SrtA peptide substrate. The fluorescence‐based SrtA detection can be achieved using either a fluorometer or a confocal microscope. A pSiRM array modified with SrtA and MMP peptide substrates was prepared to demonstrate selective and multiplexed biomarker detection. These results confirm that the fluorogenic peptide‐modified pSiRM sensing platform holds strong potential for further development as a point‐of‐care device for the management of chronic wounds.

## 4 Experimental Section

*Materials*: All pSiRM samples were prepared from phosphorus doped n‐type Si wafers, (100)‐oriented, 0.008–0.02 Ω cm resistivity (Siltronix). The Si wafers were cut into a size of 3–4 cm^2^ using a diamond cutter. All chemicals were purchased from Sigma‐Aldrich unless otherwise stated. High purity solvents (methanol, ethanol, acetone, and dichloromethane) were purchased from Chem Supply. The *S. aureus* SrtA enzyme with the molecular weight of 27.1 kDa was purchased from BPS Bioscience. The SrtA peptide substrate, Dnp‐LPETG‐(K‐FITC)‐NH_2_, was synthesized by Mimotopes Ltd Pty, Melbourne, Australia. The MMP substrate, Dabcyl‐Gaba‐Pro‐Gln‐Gly‐Leu‐Glu(EDANS)‐Ala‐Lys‐NH_2_, was purchased from Merck. Cold filterable tryptone soya broth (Oxoid) and nutrient agar (Oxoid) were purchased from ThermoFisher Scientific Australia and prepared as per the manufacturer's instructions.

*Design, Fabrication and Characterization of the pSiRM Sensing Platform*: The pSiRM samples were fabricated in a Teflon‐based electrochemical etching cell. The back of the Si wafer was contacted with an aluminum sheet and a platinum mesh was used as a cathode. A current source meter (Keithley 2425, USA) was connected to these electrodes. The electrochemical etching solution contains 25:200:1 volume ratio of hydrofluoric acid (48%, Scharlau)/water/surfactant (NCW1001, Wako Pure Chemical Industries).[[qv: 14b]] The parasitic layer of Si wafer was removed by anodically etching Si wafer at a current density of 40 mA cm^−2^ for 30 s, followed by a high current density etch of 250 mA cm^−2^ for 6 s which led to surface electropolishing. The electropolished surface was exposed to MilliQ water for 1 min to remove the sacrificial layer, then washed with methanol, acetone, dichloromethane and dried under a stream of nitrogen gas.

The pSiRM structure used in this study had a configuration of (HP/LP)_3_(HP)_4_(LP/HP)_3_. The alternating layers of different porosity were fabricated by anodically etching the pretreated Si wafer at 50 mA cm^−2^ for 2568 ms and at 25 mA cm^−2^ for 2237 ms, corresponding to HP and LP layers, respectively. The defect layer was etched at current density of 50 mA cm^−2^ for 10272 ms. The pSiRM was optically characterized using reflectance spectroscopy. The morphology of the resulting structure was characterized by scanning electron microscopy (SEM), using a Quanta 450 field emission gun (FEG) environmental SEM fitted with a solid‐state detector (SSD) and an accelerating voltage of 30 kV.

*Surface Modification and Characterization*: The freshly etched pSiRM samples were thermally hydrosilylated using neat undecylenic acid in a glass reaction flask. The neat undecylenic acid was purged with argon for 15 min to remove any oxygen. The freshly etched pSiRM samples were then immersed in undecylenic acid and purged for a further 30 min. The glass reaction flask was immersed in an oil bath at 120 °C. The reaction was performed for 3 h under argon flow. Afterward, the hydrosilylated pSiRM samples were removed from the flask, rinsed with ethanol and dried under a stream of nitrogen gas.

The hydrosilylated pSiRM samples were activated by reacting with N‐hydroxysuccinimide (NHS, Sigma‐Aldrich) (5 × 10^−3^ m) in water in the presence of 1‐(3‐dimethylaminopropyl)‐3‐ethylcarbodiimide (EDC, Fluka) (5 × 10^−3^ m) to form an NHS ester‐terminated surface. The reaction was conducted for 20 min at room temperature in a horizontal shaker. Afterward, the activated pSiRM samples were rinsed with water and dried under a stream of nitrogen gas.

The SrtA peptide substrate was immobilized on the activated pSiRM surface by overnight incubation of 1 × 10^−3^ m SrtA substrate in 50 × 10^−3^ m Hepes buffer (pH 7.4) containing 150 × 10^−3^ m NaCl, 5 × 10^−3^ m CaCl_2_, and 5 × 10^−3^ m glycine (Gly) (Hepes buffer). Afterward, the surface was rinsed with water, 2:1 water/ethanol, 1:2 water/ethanol and ethanol and then dried under a stream of nitrogen gas. Before using as a sensing platform, the modified surface was blocked with 1 m ethanolamine pH 8.5 for 10 min to prevent unspecific binding. This SrtA peptide‐modified pSiRM was then used as the pSiRM sensing platform during the sensing experiments.

Fourier‐transform infrared spectroscopy (FTIR) was used to characterize the surface after each step of the surface modification. All FTIR samples were prepared from p‐type Si wafer with a resistivity of 0.00055–0.001 Ω cm etched at a current density of 56 mA cm^−2^ for 2 min. The FTIR spectra were obtained using a Vertex 70 Hyperion microscope (Bruker) in the attenuated total reflection (ATR) mode, recorded over the range of 650–4000 cm^−1^, at a resolution of 22 cm^−2^, an aperture size of 3 mm and averaging 64 scans. The baseline was corrected and normalized with OPUS 7.2 Spectroscopy Software (Bruker).

*Biosensor Experiments*: The SrtA peptide‐modified pSiRM sensing platform was incubated with SrtA enzyme at varying concentration (4.6 × 10^−8^ m to 4.6 × 10^−18^ M) at 30 °C^23^, ^26^ for different incubation time (0, 5, 10, 15, 30, and 60 min) and then rinsed with water, 2:1 water/ethanol, 1:2 water/ethanol and ethanol. The pSiRM sensing platform was then dried under a stream of nitrogen gas and placed in a fluorometer cuvette with a sample holder to support the surface. In the fluorometer (Parkin Elmer LS 55 Luminescence Spectrometer), the pSiRM surface was placed at an angle of 36° in respect to the surface normal.^12^ The fluorescence emission was measured over a wavelength range of 500–600 nm at a fixed excitation wavelength of 495 nm, excitation and emission slit width of 5 nm each and a scan speed of 200 nm min^−1^.

*Biosensor Experiments in Complex Biological Samples*: The biosensor experiments in complex biological samples were performed using human chronic wound fluid and bacterial culture medium. The human wound fluid was collected from The Queen Elizabeth Hospital (Adelaide, South Australia) with human ethics approval based on the ethical guidelines of the 1975 Declaration of Helsinki and was approved by the Health Service Human Research Ethics Committee and the Central Northern Adelaide Health Service Ethics of Human Research Committee.

*S. aureus* was streak‐plated on nutrient agar plates and incubated at 37 °C to obtain individual colonies. Individual colonies were subcultured in tryptone soya broth (TSB) as bacterial culture medium and inoculated for 0, 0.5, 1, 3, 5, and 24 h. Cell‐free culture medium was obtained by filtering the culture using a sterile 0.22 μm filter. Individual colonies were also subcultured in wound fluid and inoculated for 24 h.

During sensing, the SrtA peptide‐modified pSiRM sensing platform was incubated with the biological sample for 30 min at 30 °C. Afterward, the surface was rinsed and dried before fluorescence measurement using the same condition as described above.

*Biosensor Characterization Using Confocal Microscopy*: Confocal microscopy was used to image the fluorescence emission of the pSiRM surface. A PDMS mask was used to protect half of the hydrosilylated pSiRM surface during covalent binding of the SrtA peptide substrate. The surface was then observed using a Carl Zeiss LSM 710 confocal microscope with an EC Plan‐Neofluar 10×/0.30 M27 objective. The surface was illuminated with light of a wavelength of 495 nm to collect the fluorescence micrograph with an emission range of 510–560 nm.

*pSiRM Microarray*: We also prepared a pSiRM array to immobilize two different fluorogenic peptide substrates. The activated pSiRM surface was printed using a SciFlexarrayer – S3 Microarray (Scienion). Two rows, with five spots each, were printed with 20 nL per spot of 10 × 10^−3^ m MMP fluorogenic peptide substrate solution, and the other two rows, also printed with five spots each, were modified with 20 nL per spot of 1 × 10^−3^ m SrtA fluorogenic peptide substrate solution. This array was incubated with human chronic wound fluid and bacterial culture medium before imaging using a confocal microscope. The surface was illuminated with light of a wavelength of 495 nm to monitor the fluorescence emission at a range of 510–560 nm from the spots immobilized with SrtA substrate and at 340 nm to monitor the fluorescence emission at a range of 435–485 nm from MMP peptide substrate.

## Supporting information

As a service to our authors and readers, this journal provides supporting information supplied by the authors. Such materials are peer reviewed and may be re‐organized for online delivery, but are not copy‐edited or typeset. Technical support issues arising from supporting information (other than missing files) should be addressed to the authors.

Supplementary

## References

[1] a) A. J. M. Boulton , L. Vileikyte , G. Ragnarson‐Tennvall , J. Apelqvist , Lancet 2005, 366, 1719; 16291066 10.1016/S0140-6736(05)67698-2

[2] K. Kirketerp‐Møller , P. Ø. Jensen , M. Fazli , K. G. Madsen , J. Pedersen , C. Moser , T. Tolker‐Nielsen , N. H. Hoiby , M. Givskov , T. Bjarnsholt , J. Clin. Microbiol. 2008, 46, 2717. 18508940 10.1128/JCM.00501-08 PMC2519454

[3] T. Bjarnsholt , K. Kirketerp‐Møller , P. Ø. Jensen , K. G. Madsen , R. Phipps , K. Krogfelt , N. H. Hoiby , M. Givskov , Wound Rep. Reg. 2008, 16, 2. 10.1111/j.1524-475X.2007.00283.x 18211573

[4] A. W. Maresso , O. Schneewind , Pharmacol. Rev. 2008, 60, 128. 18321961 10.1124/pr.107.07110

[5] a) M. J. J. B. Sibbald , A. K. Ziebandt , S. Engelmann , M. Hecker , A. de Jong , H. J. M. Harmsen , G. C. Raangs , I. Stokroos , J. P. Arends , J. Y. F. Dubois , J. M. van Dijl , Microbiol. Mol. Biol. Rev. 2006, 70, 755; 16959968 10.1128/MMBR.00008-06 PMC1594592

[6] R. P. Novick , Mol. Microbiol. 2003, 48, 1429. 12791129 10.1046/j.1365-2958.2003.03526.x

[7] G. Brackman , L. De Meyer , H. J. Nelis , T. Coenye , J. Appl. Microbiol. 2013, 114, 1833. 23490006 10.1111/jam.12191

[8] a) K.‐B. Oh , K.‐W. Nam , H. Ahn , S. J. S. Kim , W. Mar , Biochem. Biophys. Res. Commun. 2010, 396, 440; 20433810 10.1016/j.bbrc.2010.04.113

[9] I.‐M. Jonsson , S. K. Mazmanian , O. Schneewind , T. Bremell , A. Tarkowski , Microb. Infect. 2003, 5, 775. 10.1016/s1286-4579(03)00143-6 12850203

[10] a) H. Ton‐That , O. Schneewind , J. Biol. Chem. 1999, 274, 24316; 10446208 10.1074/jbc.274.34.24316

[11] a) E. M. Purcell , Phys. Rev. 1946, 69, 681;

[12] F. S. H. Krismastuti , S. Pace , N. H. Voelcker , Adv. Funct. Mater. 2014, 24, 3639.

[13] a) H. J. Kang , F. Coulibaly , T. Proft , E. N. Baker , PLoS One 2011, 6, e15969; 21264317 10.1371/journal.pone.0015969 PMC3019223

[14] a) L. A. DeLouise , P. M. Kou , B. L. Miller , Anal. Chem. 2005, 77, 3222; 15889912 10.1021/ac048144+

[15] S. Chan , P. M. Fauchet , Y. Li , L. J. Rothberg , B. L. Miller , Phys. Status Solidi A 2000, 182, 541.

[16] L. A. DeLouise , P. M. Fauchet , B. L. Miller , A. A. Pentland , Adv. Mater. 2005, 17, 2199.

[17] H. Ouyang , P. M. Fauchet , Proc. SPIE, 2005, 6005, 600508.

[18] a) K. A. Kilian , T. Bocking , J. J. Gooding , Chem. Commun. 2009, 630; 10.1039/b815449j 19322406

[19] a) J. M. Buriak , M. P. Stewart , T. W. Geders , M. J. Allen , H. C. Choi , D. Raftery , L. T. Canham , J. Am. Chem. Soc. 1999, 121, 11491;

[20] T. Bocking , K. A. Kilian , K. Gaus , J. J. Gooding , Adv. Funct. Mater. 2008, 18, 3827.

[21] B. Sciacca , F. Frascella , A. Venturello , P. Rivolo , E. Descrovi , F. Giorgis , F. Geobaldo , Sens. Actuators B 2009, 137, 467.

[22] G. Palestino , V. Agarwal , R. Aulombard , E. Perez , C. Gergely , Langmuir 2008, 24, 13765. 18959435 10.1021/la8015707

[23] S. K. Mazmanian , G. Liu , H. Ton‐That , O. Schneewind , Science 1999, 285, 760. 10427003 10.1126/science.285.5428.760

[24] S. P. Askin , P. M. Schaeffer , Analyst 2012, 137, 5193. 23001025 10.1039/c2an35857c

[25] Y. Yarets , L. Rubanov , I. Novikova , N. Shevchenko , EWMA J. 2013, 13, 7.

[26] B. C. Nwomeh , H.‐X. Liang , I. K. Cohen , D. R. Yager , J. Surg. Res. 1998, 81, 189. 10.1006/jsre.1998.5495 9927539

[27] F. S. H. Krismastuti , A. J. Cowin , S. Pace , E. Melville , T. R. Dargaville , N. H. Voelcker , Aust. J. Chem. 2013, 66, 1428.

