# Supplementary material for: Toward Multiplexing Detection of Wound Healing Biomarkers on Porous Silicon Resonant Microcavities
Source: Adv Sci (Weinh). 2016 Feb 4;3(6):1500383. doi: 10.1002/advs.201500383 (PMC5067563; doi:10.1002/advs.201500383)
Supplement: Supplementary file 1 — Supplementary [file ADVS-3-0b-s001.pdf]

Copyright WILEY-VCH Verlag GmbH & Co. KGaA, 69469 Weinheim, Germany, 2016.

ADVANCED SCIENCE Open Access

Supporting Information

for *Adv. Sci.*, DOI: 10.1002/advs.201500383

Toward Multiplexing Detection of Wound Healing Biomarkers on Porous Silicon Resonant Microcavities

*Fransiska Sri Herwahyu Krismastuti, Alex Cavallaro, Beatriz Prieto-Simon, and Nicolas H. Voelcker**

Copyright WILEY-VCH Verlag GmbH & Co. KGaA, 69469 Weinheim, Germany, 2013.

Supporting Information

Towards Multiplexing Detection of Wound Healing Biomarkers on Porous Silicon Resonant Microcavities

*Fransiska Sri Herwahyu Krismastuti, Alex Cavallaro, Beatriz Prieto-Simon, and Nicolas H. Voelcker**

Figure S1.

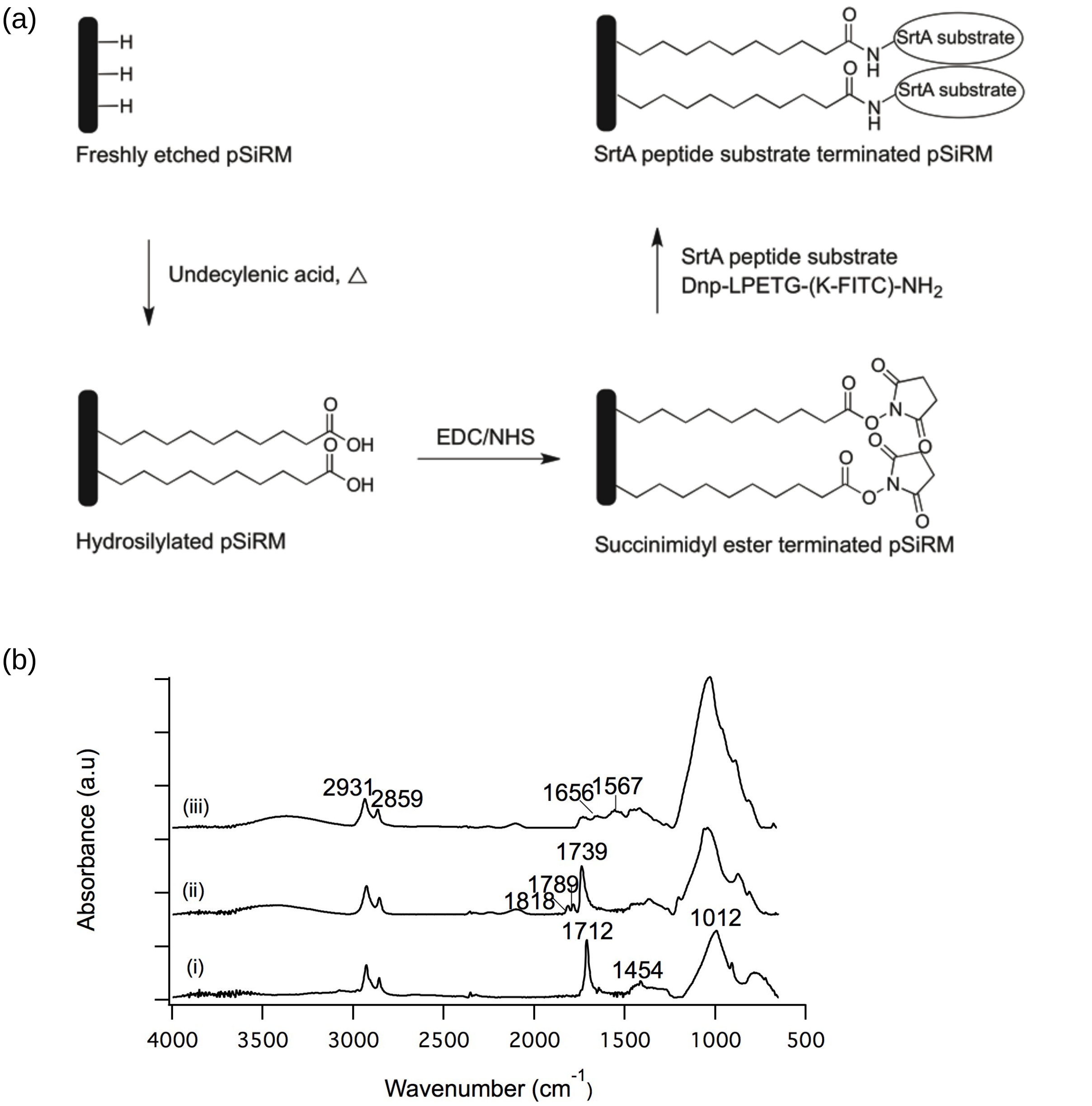

Figure S1. (a) Schematic of surface modification reaction and (b) FTIR-ATR spectra (baseline corrected) of the pSiRM surface after (i) hydrosilylation with undecylenic acid, (ii) activation with EDC/NHS and (iii) immobilization of the fluorogenic SrtA peptide substrate.

The thermal hydrosilylation reaction led to the disappearance of the Si–H signals and the appearance of characteristic bands at 1454 cm^{-1}, 2859 cm^{-1} and 2931 cm^{-1} corresponding to the $\Delta_{CHtet.}$ deformation mode of methylenes and the stretching vibrational of aliphatic C–H bonds, respectively (Spectrum (i)). The band at 1712 cm^{-1} is characteristic for the $\nu_{(C=O)}$ stretching mode of a carboxylic acid. The band at 1012 cm^{-1} attributed to the Si–O stretching vibrational indicates the presence of silicon dioxide at the pSiRM surface. This is commonly observed in the hydrosilylated pSi surface.[1]

The activation of the carboxylic acid terminated layer with EDC/NHS (Spectrum (ii)) was observed in the spectral changes including a triplet band at 1739 cm^{-1}, 1789 cm^{-1} and 1818 cm^{-1} which is characteristic for the formation of the NHS ester group. The first two bands, 1739 cm^{-1} and 1789 cm^{-1} were assigned to the $\nu_{as(C=O)}$ antisymmetric stretching vibrational mode and to the $\nu_{s(C=O)}$ symmetric stretching vibration of the succinimidyl cycle, respectively, while the band at 1818 cm^{-1} corresponds to the $\nu_{s(C=O)}$ symmetric stretching vibrational mode and the $\nu_{(C=O)}$ stretching vibrational mode of the succinimidyl ester carbonyl.[1a, 1c] After immobilization of the SrtA peptide substrate (Spectrum (iii)), the bands at 1656 cm^{-1} and 1567 cm^{-1} attributed to the amide I and amide II bonds appeared. These bands confirm that the SrtA peptide substrate was covalently attached to the pSiRM surface via amide bonds.[1a]

Figure S2.

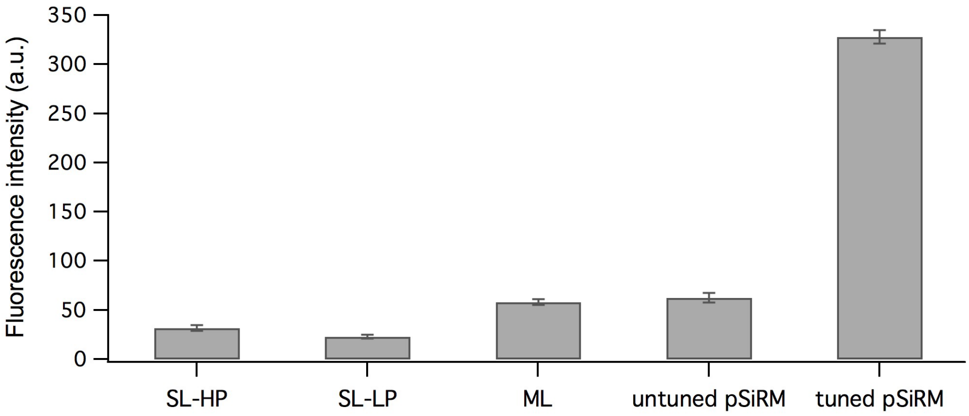

Figure S2. Fluorescence intensity from the fluorogenic SrtA peptide substrate-modified pSiRM after incubation with SrtA in Hepes buffer at different pSi structure: single layer with HP (SL-HP), single layer with LP (SL-LP), multilayer pSi (ML), untuned pSiRM with the microcavity dip at 520 nm and tuned pSiRM with the microcavity dip at 514 nm.

The results in **Figure S2** show that the highest fluorescence emission was obtained from the tuned pSiRM confirming the fluorescence enhancement effect of microcavity.

Figure S3.

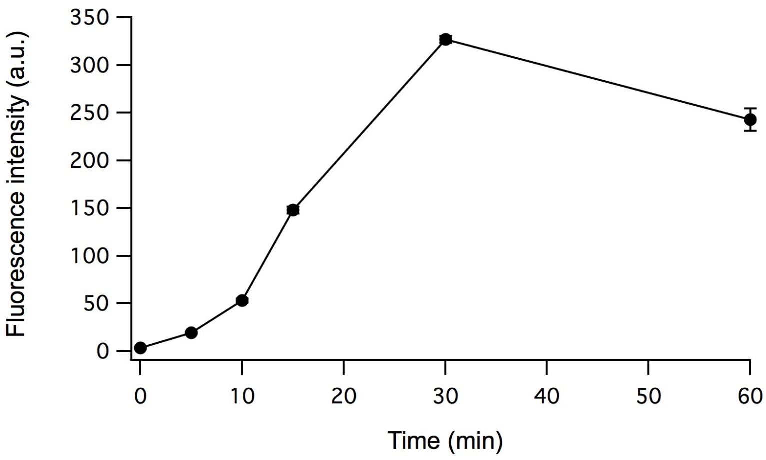

Figure S3. Fluorescence intensity from the fluorogenic SrtA peptide substrate-modified pSiRM after incubation with SrtA in Hepes buffer at different times. Error bars were calculated from three independent experiments.

The graph shows that at SrtA concentration of 4.6 x 10^{-8} M, after 5 min incubation time, a conspicuous fluorescence emission signal appeared, indicating that the SrtA substrate was already being cleaved. The fluorescence intensity signal increased with incubation time up to 30 min. The signal then decreased, possibly due to fluorescence self-quenching, which can occur as a result of non-radiative energy transfer between fluorescence molecules in close proximity.[2] Interestingly, another study also observed maximum emission after 30 min interaction with SrtA.[3]

Figure S4.

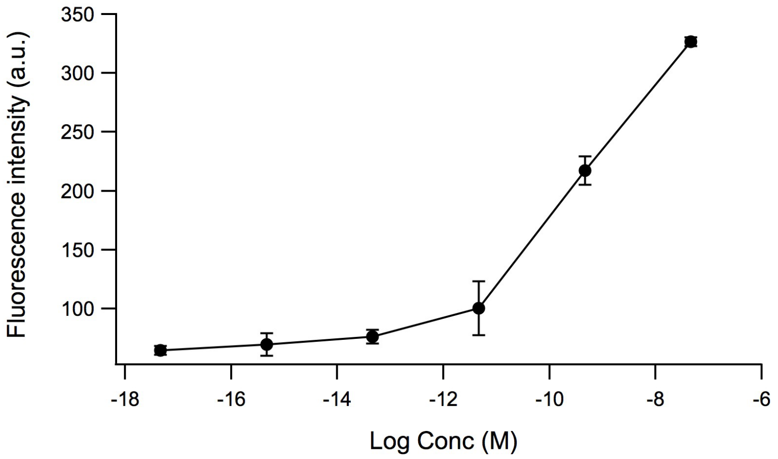

Figure S4. Fluorescence intensity from the fluorogenic SrtA peptide substrate-modified pSiRM after incubation with different concentrations of SrtA enzyme in Hepes buffer. Error bars were calculated from three independent experiments.

The dependence of fluorescence intensity on different SrtA enzyme concentrations is presented in Figure S4. This figure shows that optical signal increased linearly with increasing concentration of SrtA enzyme from 4.6 x 10^{-12} M to 4.6 x 10^{-8} M (four orders of magnitude) with a linear regression equation of y = 57 x + 744 (R^2 = 0.9997). At lower SrtA concentrations, increasing the concentration of SrtA only gradually increased the fluorescence signal. From the experiments, the lowest concentration tested was 4.6 x 10^{-18} M. The limit of detection (LOD) and the limit of quantitation (LOQ) were calculated as y_b + 3Std_b and y_b + 10 Std_b, respectively, where y_b is the fluorescence measured for the blank (control solution in the absence of SrtA) and Std_b is the standard deviation of blank. The LOD and LOQ were calculated based on the linear range of the various concentrations tested (from 4.6 x 10^{-12} to 4.6 x 10^{-8} M). Using those equations, the calculated LOD and LOQ were 8.0 x 10^{-14} M and 8.3 x 10^{-14} M, respectively.

Figure S5.

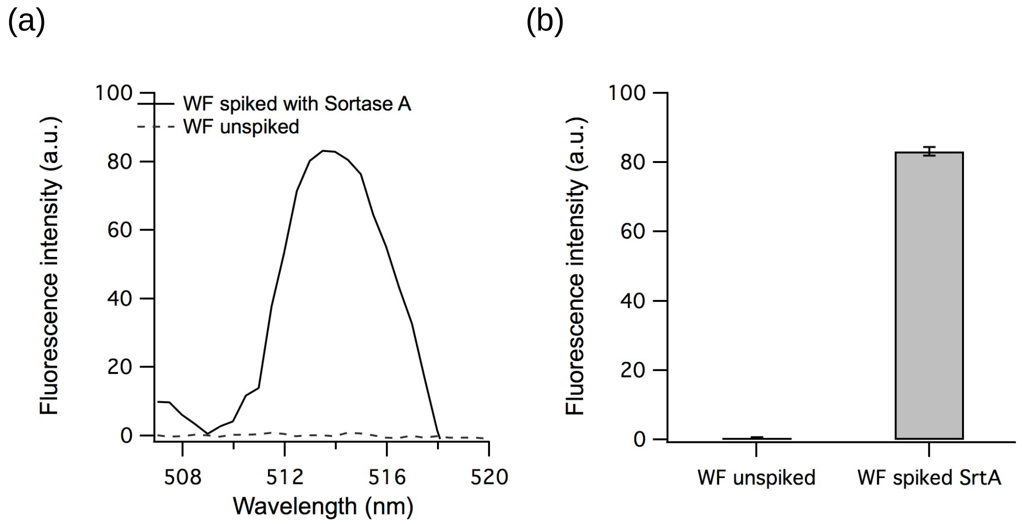

Figure S5. (a) Fluorescence intensity from the SrtA fluorogenic peptide substrate-modified pSiRM after incubation with wound fluid (dashed line) and wound fluid spiked with 4.6 x 10-

^{12} M SrtA (full line) (b) The bar charts showing the fluorescence intensity for unspiked and spiked wound fluid with error bars calculated from three independent experiments.

Figure S5 shows the fluorescence signal from FITC after 30 min incubation of wound fluid (left bar) and wound fluid spiked with SrtA (right bar) on the pSiRM sensor. No emission peak was seen for the unspiked wound fluid, suggesting that the human wound fluid sample used in this study did not contain any SrtA. However, upon incubation with the SrtA-spiked wound fluid sample, the fluorescence intensity emitted from the surface was 83 ± 1 a.u. **(Figure S5(b))** giving a SrtA concentration of 2.2 x 10^{-12} ± 1.1 x 10^{-13} M if interpolated into the linear range of the graph in **Figure S4**. This result indicates that the sensing platform could detect the presence of SrtA spiked in complex biological matrices.

Figure S6.

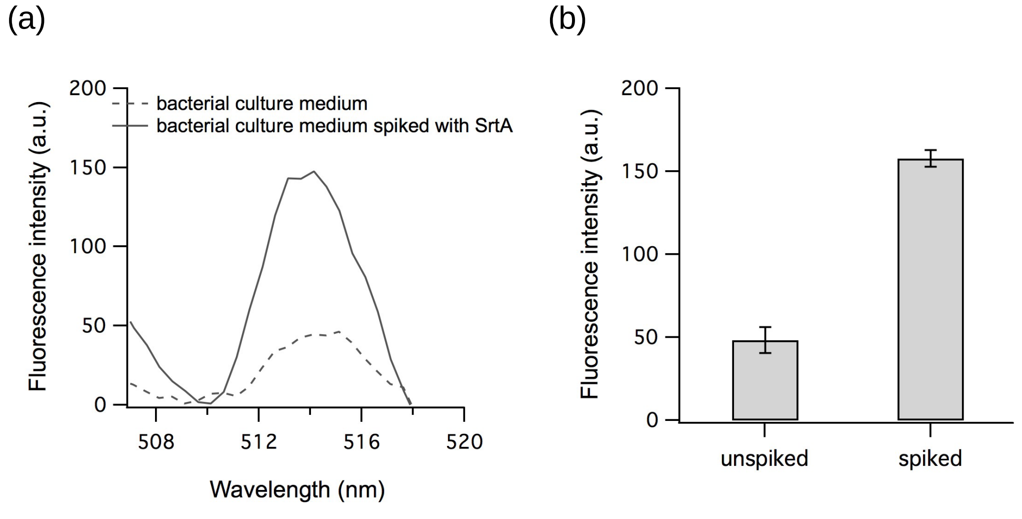

Figure S6. (a) Fluorescence intensity from the SrtA fluorogenic peptide substrate-modified pSiRM after incubation with the bacterial culture medium inoculated for 1 h (dashed line) and after being spiked with 4.6 x 10^{-12} M SrtA (full line) (b) The bar chart showing the

fluorescence intensity for unspiked and spiked bacterial culture medium with error bars calculated from three independent experiments.

A sensing experiment was also performed for *S. aureus* bacterial culture medium, which had been inoculated with *S. aureus*. We first tested the medium after 1 h of inoculation. After incubating the bacterial culture medium with the SrtA peptide substrate-modified pSiRM sensing platform, a strong fluorescence emission was observed at 514 nm (48 ± 8 a.u.) (dashed line in **Figure S6(a)** and left bar in **Figure S6(b)**). This fluorescence signal indicates cleavage of the substrate peptide.

To confirm that the observed signal was due to the presence of SrtA enzyme in the bacterial culture medium, we spiked the medium with 4.6×10^{-12} M SrtA. This increased the fluorescence intensity to 158 ± 5 a.u. (full line in **Figure S6(a)** and right bar in **Figure S6(b)**). This increase in intensity (109 ± 3 a.u. higher than the intensity measured for the unspiked bacterial culture medium) gave a SrtA concentration of $6.6 \times 10^{-12} \pm 8.5 \times 10^{-14}$ M if interpolated into the linear range from the graph in **Figure S4**.

References

[1] a) T. Bocking, K. A. Kilian, K. Gaus, J. J. Gooding, *Adv. Funct. Mater.* **2008**, *18*, 3827-3833; b) R. Boukherroub, J. T. C. Wojtyk, D. D. M. Wayner, D. J. Lockwood, *J. Electrochem. Soc.* **2002**, *149*, H59-H63; c) S. Sam, L. Touahir, J. Salvador Andresa, P. Allongue, J. N. Chazalviel, A. C. Gouget-Laemmel, C. Henry de Villenueve, A. Moraillon, F. Ozanam, N. Gabouze, S. Djebbar, *Langmuir* **2010**, *26*, 809-814; d) B. Sciacca, E. Secret, S. Pace, P. Gonzalez, F. Geobaldo, F. Quignard, F. Cunin, *J. Mater. Chem.* **2011**, *21*, 2294.

[2] C. Deka, B. E. Lehnert, N. M. Jones, L. A. Sklar, J. A. Steinkamp, *Cytometry* **1996**, *25*, 271-279.

[3] S. K. Mazmanian, G. Liu, H. Ton-That, O. Schneewind, *Science* **1999**, *285*, 760-763.
